# A novel, automated method for measuring mitral valve annular velocity from standard cine TrueFISP data - a feasibility study

**DOI:** 10.1186/1532-429X-13-S1-O48

**Published:** 2011-02-02

**Authors:** Peter J Weale, Christoph Guetter, Jeremy D Collins, Marie Wasielewski, Neil Chatterjee, Marie-Pierre Jolly, Hui Xue, Xiaoguang Lu, Jens Guehring, Sven Zuehlsdorff, James Carr

**Affiliations:** 1 Siemens Healthcare USA, Chicago, IL, USA; 2 Siemens Corporate Research, Princeton, NJ, USA; 3 Northwestern University, Department of Radiolgy, Chicago, IL, USA; 4 Northwestern University Feinberg School of Medicine, Chicago, IL, USA

## Introduction

A novel method for determining a correlate of Tissue Doppler Imaging (TDI) parameters from cine TrueFisp data. Using the velocity of automatically identified and tracked landmarks,time/velocity data and hence s', e' and a' can be derived.

## Purpose

To validate mitral valve annular velocities extracted in an automated fashion using an established algorithm for database guided landmark detection and temporal propagation based on inverse consistent non-rigid registration [1] in a normal population.

To correlate the derived early diastolic myocardial peak-velocity measurements (e prime or e') against phase contrast methods and to evaluate correlation with age.

## Methods

**Figure 1:**
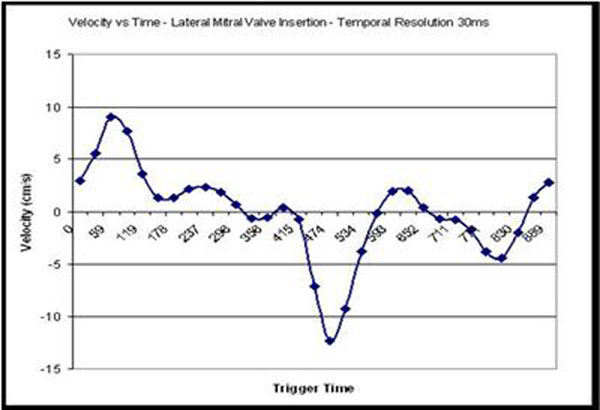
Time/Velocity curve showing depiction of s' e' and a'

A cohort of 12 healthy volunteers (7F, 22 to 62 yrs) was recruited under an IRB approved protocol. Cine TrueFISP data was acquired in the four chamber orientation with temporal resolution of 7,10,20,30,40 and 50ms. with the aim of assessing the performance of the automatic tracking algorithm with increased sampling rate. e' was derived from time-velocity curves (Figure 1) extracted from the frame to frame change in position of the track points.

**Figure 2:**
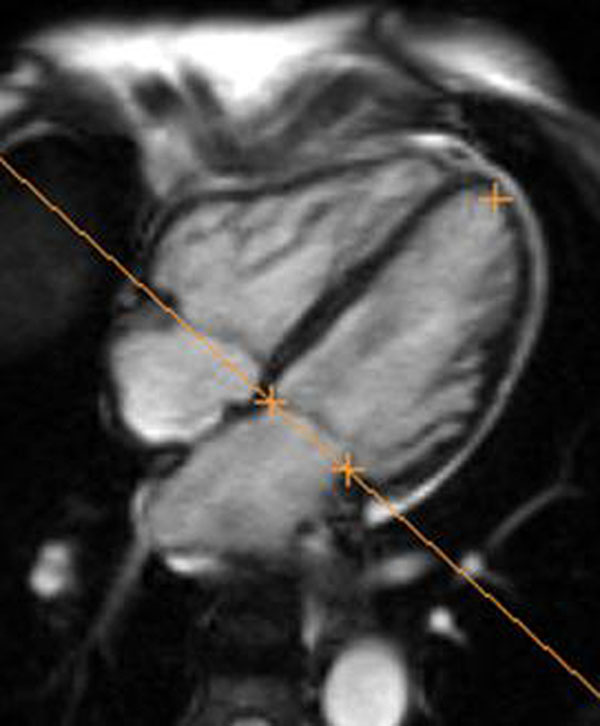
Automatically generated tracking points

In a sub-cohort of 6 subjects breath-hold and free breathing phase contrast data (Venc 25cm/s) was acquired in the short axis orientation at a slice position where the myocardium on the apical side of the valve ring was within the slice throughout the cardiac cycle.

Lateral and septal myocardium at the intersection of the four chamber orientation was then evaluated using standard flow post-processing and e' velocities derived.

## Results

For each temporal resolution, the mean e' velocity was calculated across all subjects. The results demonstrate the expected increase in detected peak e' with improving temporal resolution but at <20ms the data develops an increasing variance as illustrated in table 1.

**Table 1:** Lateral and Septal e prime - effect of temporal resolution

Temporal Resolution(ms)	Septal e' cm.s^-1^	Septal Standard Deviation	Lateral e' cm.s^-1^	Lateral Standard Deviation
7	46.69	29.81	28.69	17.91
10	26.24	17.83	18.62	8.02
20	15.48	9	14.41	5.7
30	10.72	5.81	10.72	5.81
40	8.45	4.1	8.45	4.1
50	6.37	2.6	6.37	2.6

Using e' derived from the lateral landmark with 30 ms temporal resolution , a good correlation with both breath-hold and free breathing phase contrast data (*r* = 0.80 for both) is seen.

Measured e' demonstrates an inverse correlation with subject age (*r =* - 0.74)

## Conclusions

This novel method of deriving mitral annular velocities is potentially a method for extracting data which may correlate with TDI.

It is simple extension of an existing algorithm which provides measurement of s' eand a' from standard imaging data. Validation against TDI is ongoing.
